# Chimeric Protein IPath^®^ with Chelating Activity Improves Atlantic Salmon’s Immunity against Infectious Diseases

**DOI:** 10.3390/vaccines9040361

**Published:** 2021-04-09

**Authors:** Valentina Valenzuela-Muñoz, Bárbara P. Benavente, Antonio Casuso, Yeny Leal, Cristian Gallardo-Escárate

**Affiliations:** 1 Interdisciplinary Center for Aquaculture Research (INCAR), University of Concepción, Concepción 4030000, Chile; valevalenzuela@udec.cl (V.V.-M.); bbenaventec@gmail.com (B.P.B.); acasuso@udec.cl (A.C.); yleal@udec.cl (Y.L.); 2 Laboratory of Biotechnology and Aquatic Genomics, Department of Oceanography, University of Concepción, Concepción 4030000, Chile

**Keywords:** iron transport, chimeric protein, chelating activity, *Salmo salar*, *Aeromonas salmonicida*, *Caligus rogercresseyi*, *Piscirickettsia salmonis*

## Abstract

Infection processes displayed by pathogens require the acquisition of essential inorganic nutrients and trace elements from the host to survive and proliferate. Without a doubt, iron is a crucial trace metal for all living organisms and also a pivotal component in the host–parasite interactions. In particular, the host reduces the iron available to face the infectious disease, increasing iron transport proteins’ expression and activating the heme synthesis and degradation pathways. Moreover, recent findings have suggested that iron metabolism modulation in fish promotes the immune response by reducing cellular iron toxicity. We hypothesized that recombinant proteins related to iron metabolism could modulate the fish’s immune system through iron metabolism and iron-responsive genes. Here a chimeric iron transport protein (IPath^®^) was bioinformatically designed and then expressed in a recombinant bacterial system. The IPath^®^ protein showed a significant chelating activity under in vitro conditions and biological activity. Taking this evidence, a vaccine candidate based on IPath^®^ was evaluated in Atlantic salmon challenged with three different fish pathogens. Experimental trials were conducted using two fish groups: one immunized with IPath^®^ and another injected with adjutant as the control group. After 400 accumulated thermal units (ATUs), two different infection trials were performed. In the first one, fish were infected with the bacterium *Aeromonas salmonicida*, and in a second trial, fish were exposed to the ectoparasite *Caligus rogercresseyi* and subsequently infected with the intracellular bacterium *Piscirickettsia salmonis*. Fish immunized with IPath^®^ showed a significant delay in the mortality curve in response to *A. salmonicida* and *P. salmonis* infections. However, no significant differences between infected and control fish groups were observed at the end of the experiment. Notably, sea lice burden reduction was observed in vaccinated Atlantic salmon. Transcriptional analysis evidenced a high modulation of iron-homeostasis-related genes in fish vaccinated with IPath^®^ compared to the control group during the infection. Moreover, increasing expression of Atlantic salmon *IgT* was associated with IPath^®^ immunization. This study provides evidence that the IPath^®^ protein could be used as an antigen or booster in commercial fish vaccines, improving the immune response against relevant pathogens for salmon aquaculture.

## 1. Introduction

Iron is an essential element for most eukaryotic and prokaryotic organisms, with an essential role in hemoglobin synthesis, oxygen transport, energy production, DNA synthesis, cellular respiration, as well as being a cofactor of a series of enzymes involved in the maintenance of cellular integrity [1,2,3]. An organism’s iron availability is regulated by a series of proteins with a high affinity to iron transport and storage. For instance, ferritin is a multimeric protein highly conserved among species that stores up to 4000 iron atoms [4,5]. Ferritin exhibits 24 subunits of 200 amino acids and comprises two subunits, the heavy (H) and light (L) chains of 21 and 19 kDa, respectively [4,5]. Ferritin synthesis is regulated by iron regulatory proteins (IRPs) according to the system’s available iron concentration [6,7]. Among iron transport proteins, the transferrin shows chelating activity, and it is expressed in eukaryotes’ serum where the Fe^+3^ is maintained in a redox-inert state. Transferrin also plays a role in fish immunity by reducing iron available for the microorganisms [5,8]. Moreover, hepcidin is an oligopeptide with antibacterial activity and a primary iron regulation protein [9,10].

Different studies suggest an immune role of iron-homeostasis-related proteins [11]. Host iron limitation is a strategy to reduce the iron available, reducing the pathogen’s success [12]. In fish, it is reported that there are changes in iron-homeostasis-related genes during infections. For instance, sea bass has been reported to increase *hepcidin* gene expression levels in fish liver infected with *Photobacterium damselae* [13]. Ferritin gene overexpression has been observed in the sea bass brain under bacterial infection [5]. Moreover, in turbot, an upregulation of the *ferritin* gene after microbial challenge has been reported [14]. A study performed in sea bass reported a downmodulation of transferrin in the liver and brain during bacterial infection, while ferritin transcripts were overexpressed [5]. Moreover, in Atlantic salmon infected with the bacteria *Piscirickettsia salmonis*, high expression levels of iron transport proteins, such as ferritin, haptoglobin, hepcidin, and transferrin receptor, have been evidenced [15]. Furthermore, Atlantic salmon infected with the ectoparasite *Caligus rogercresseyi* increase the ferritin expression levels in response to sea louse infection [16,17]. 

Furthermore, iron-homeostasis-related proteins have demonstrated effects on pathogen viability. For instance, inhibition of the white spot syndrome virus (WSSV) replication has been reported in shrimp injected with recombinant ferritin [18]. The ferritin knockdown in *Procambarus clarkii* also promotes the replication of WSSV [19]. *Listonella anguillarum* growth inhibition in turbot has also been reported [20]. Moreover, the antiviral activity of two isoforms of hepcidin has been reported in turbot [21]. On the other hand, immunosuppression effects have been reported in European sea bass larvae exposed to recombinant ferritin during *Vibrio anguillarum* infection [22]. Moreover, for terrestrial organisms, recombinant ferritin has been used for ectoparasite control [23]. 

Chilean salmon aquaculture is affected by several pathogens, including viruses, bacteria, and ectoparasites. One of the most prevalent is the ectoparasite copepod *C. rogercresseyi*, commonly named as sea louse. Lice infection produces skin damage in infected fish, facilitating other opportunistic pathogens [24]. Molecular studies have highlighted the various immune response mechanisms in play during *C. rogercresseyi* infestation, such as an increase in inflammatory cytokines, iron regulation, protease secretion, immunoglobulin levels, and Th1 and Th2 responses [25,26]. The intracellular bacterium, *Piscirickettsia salmonis,* is the etiological agent of salmonid rickettsial septicemia (SRS). It is considered responsible for 60% to 80% of the mortalities produced by infectious diseases in salmonid species farming [27]. The bacterium infects and replicates in macrophages, stimulating the innate immune response and oxidative defense response [28]. *P. salmonis* also modulates the pro-inflammatory cytokine response, the iron regulation, cytoskeletal reorganization, and protein transportation to evade the host’s immune response [29,30]. Another pathogen is *Aeromonas salmonicida*, which causes ulcerative disease and furunculosis and is the main cause of mortality occasioned by extracellular bacteria in Atlantic salmon [31,32]. Molecular studies have showed higher expression of pro-inflammatory genes, such as *TLR5M, TLR5S, GATA3, IFN-γ, IL-17D*, as well as the pleiotropic cytokine gene *IL-10* in infected Atlantic salmon [33]. Interestingly, recent findings have showed an intraerythrocytic phase of *A. salmonicida* during the infection [34]. Overall, the importance of Atlantic salmon iron homeostasis regulation is well reported during these pathogens’ infection [15,17,35,36]. This study aimed to evaluate a chimeric protein named IPath^®^ as a candidate vaccine against prevalent pathogens in Chilean salmon farming. 

## 2. Materials and Methods 

### 2.1. Design and Expression of a Recombinant Chimeric Protein (IPath^®^)

The chimeric construct (IPath^®^) is containing residues from the iron-binding domain of Atlantic salmon transferrin (GenBank: AAA18838) and Atlantic salmon ferroxidase diiron center of ferritin subunit H (GenBank: NP_001139960). The sequences were bioinformatically analyzed to concatenate a unique sequence named IPath^®^. The nucleotide sequence of IPath^®^ was synthesized in GenScript with codon optimization and cloned into the expression vector pET30a (+), which adds a C-terminal His-tag. The IPath^®^ was transformed in *Escherichia coli* BL21, and the chimeric protein expression was induced with 1 mM of isopropyl-D-1-thiogalactopyranoside (IPTG) (Invitrogen, Carlsbad, CA, USA) in Luria-Bertani (LB) medium with kanamycin (50 μg/mL) (USBiological, Salem, MA, USA) for six hours at 37 °C with constant agitation. The obtained pellet was resuspended in lysis buffer (sodium phosphate 20 mM, pH 7.5). It was sonicated on ice 5 s ON, 10 s OFF, for 5 min, with an amplitude of 90%, followed by centrifugation for separating the soluble phase. The protein purification was performed by size-exclusion chromatography, using the ÄKTAprime plus (GE Healthcare Life Sciences, Boston, MA, USA) in a Superdex^TM^ 75 column (GE Healthcare Life Sciences, USA). The IPath^®^ induction was conducted in bacterial culture, and the purified protein was evaluated by SDS-PAGE (12%) under reducing conditions. Western blotting was performed using an anti-His Horseradish Peroxidase (HRP)-conjugated antibody (Thermo Fisher Scientific, Waltham, MA, USA) in a 1:2000 dilution. The protein quantification was carried out with the commercial kit BCA Protein Assay (Thermo Fisher Scientific, USA), following the manufacturer’s instructions. The results were observed in the photo-documenting system, iBright CL1000 (Invitrogen, USA).

### 2.2. IPath^®^ Iron-Chelating Activity 

The iron-chelating ability of purified IPath^®^ was determined following a previously reported method [37,38]. Briefly, 130 µL of IPath^®^ (1, 3, 6, 9, 12 µg/mL) was added to 50 µL solution of 0.5 mM FeCl_2_ (Sigma-Aldrich, Saint Louis, MO, USA) and incubated for 10 min at room temperature with shaking. The reaction was initiated by adding 120 µL of 0.5 mM of ferrozine (Sigma-Aldrich, USA). IPath^®^ iron catching was measured by forming a ferrous iron–ferrozine complex at 562 nm using the spectrophotometer MULTISCAN GO (Thermo Fisher Scientific, USA). A minimum of three replicates was used to record the average absorbance value for the IPath^®^ iron-chelating activity. The chelating activity was calculated as a percentage using the absorbance measures of the negative control (C, the buffer used for dialysis after purification), IPath^®^ sample (S), blank (B), into the equation (C-(S-B))/C × 100%. Additionally, 1 mM of deferoxamine mesylate (Sigma-Aldrich) was added as a positive control. 

### 2.3. Immunization and Infection Trials

Two candidate vaccines were formulated. The first one was using 30% of antigen (30 µg of IPath^®^), and 70% of adjuvant (MONTANIDE™ ISA 761VG); the second was a control formulation Phosphate-buffered saline (PBS) and adjuvant. Samples of Atlantic salmon of 90 g (n = 300) were produced from the experimental laboratory of the Marine Biological Station, University of Concepción, Dichato, Chile. Atlantic salmon were acclimatized for 15 days in seawater (13 °C) with periodic feeding. Then, 150 fish were separated into two groups and intraperitoneally injected with 100 µL of IPath^®^ formulation or 100 µL of control formulation. Each experimental fish group, composed of 25 individuals of Atlantic salmon, was evaluated in triplicate. After 400 accumulated thermal units (ATUs) or 30 days at 13 °C [39], both groups were challenged with an intraperitoneal injection of 1 × 10^9^ cells/mL in 100 µL of *A. salmonicida*. Mortality was recorded daily. Head kidney and blood cell samples were fixed in RNAlater RNA Stabilization Reagent^®^ (Ambion, Life Technologies, Carlsbad, CA, USA) and stored at −80 °C until RNA extraction. 

A second infection trial was conducted, comprising 150 individuals of Atlantic salmon infected with the sea louse *C. rogercresseyi* and subsequently infected with the intracellular bacteria *P. salmonis.* Acclimated fish were randomly placed into six tanks (500 L) and divided into two groups with 25 fish per tank or three replicates of each experimental fish group. One group was intraperitoneally injected with 100 µL of IPath^®^ formulation and the second one with 100 µL of control formulation. After 400 ATUs, fish were infected with 35 copepods per fish and fed daily for 30 days. After 25 days of infestation, adults of *C. rogercresseyi* were counted. Samples of *C. rogercresseyi* females were also collected and fixed in RNAlater (Ambion, Life Technologies, Carlsbad, CA, USA). Furthermore, head kidney tissue and blood cells of five Atlantic salmon of each group were fixed in RNAlater (Ambion, USA) for RNA isolation. Finally, to explore the vaccine’s potential impact in a secondary-pathogen scenario, individuals were infected with *P. salmonis*. Each experimental group was intraperitoneally injected with a dose of 1 × 10^8^ cells/mL in 100 µL (TCID_50_). Mortality was recorded daily. Samples of the head kidney and blood cells were collected from infected salmons at the beginning of the mortality caused by *P. salmonis* infection. All animal procedures were carried out under the guidelines approved by the Ethics Committee of the University of Concepción. The experimental design for the current study considered the replacement, reduction and refinement (3Rs) guidelines for animal testing. 

### 2.4. RNA Extraction and RT-qPCR Analysis in Atlantic Salmon Exposed to Different Pathogens

Total RNA extraction was conducted using TRIzol^®^ Reagent (Ambion, Life Technologies™, USA) following the manufacturer’s suggested protocol. The extracted RNA’s concentration and purity were determined using a Nanodrop ND-100 spectrophotometer (NanoDrop Technologies, Waltham, MA, USA). Finally, the total RNA integrity was checked by electrophoresis in denaturing MOPS gel. cDNA synthesis was performed using 200 ng/μL of initial total RNA and the RevertAid™ H Minus First Strand cDNA Synthesis Kit (Thermo Fisher Scientific™, USA) using the manufacturer’s instructions. 

RT-qPCR analysis of genes associated with iron homeostasis and immune response were performed in Atlantic salmon experimental groups (Table 1). The comparative ΔΔCt relative expression analysis method was used. The selection of the housekeeping gene for the experiment was based on evaluating the stability of the *elongation factor-α* (*EF-α*), *β-tubulin*, and *18S* genes by NormFinder. Through this, *EF-α* was selected for gene normalization. Each RT-qPCR reaction was carried out in a final volume of 10 μL using the commercial PowerUp SYBR Green Master Mix kit (Applied Biosystems, Foster City, CA, USA). The RT-qPCR reactions were performed on the StepOnePlus (Applied Biosystems, Life Technologies, Foster City, CA, USA), under the following conditions: 95 °C for 10 min, 40 cycles at 95 °C for 15 s and alignment temperature for 30 s (see Table 1), ending with 30 s at 72 °C. Additionally, *IgM* and *IgT* expression levels were measured using TaqMan probes previously described for Atlantic salmon [40]. Each reaction was carried out in a final volume of 12 μL using the commercial kit Kapa Probe Fast Universal qPCR (Kapa Biosystems, Darmstadt, DE). PCR amplification for the reaction with a TaqMan probe was performed on the StepOnePlus™ device (Applied Biosystems^®^, Life Technologies™, USA), under the following conditions: 95 °C for 10 min, 45 cycles at 95 °C for 15 s, and 60 °C for 1 min. The statistical analysis of the results obtained was carried out using the ANOVA-1 test and Student’s *t*-test in the GraphPad Prism (version 8.4.3). Additionally, two principal component analyses (PCAs) where data of iron-homeostasis- and immune-related genes as variables were used to assess the correlation between the transcriptional expression and (i) Atlantic salmon immunization and (ii) pathogen challenge.

## 3. Results

### 3.1. The Iron-Chelating Activity of Chimeric Protein

**Figure 1:**
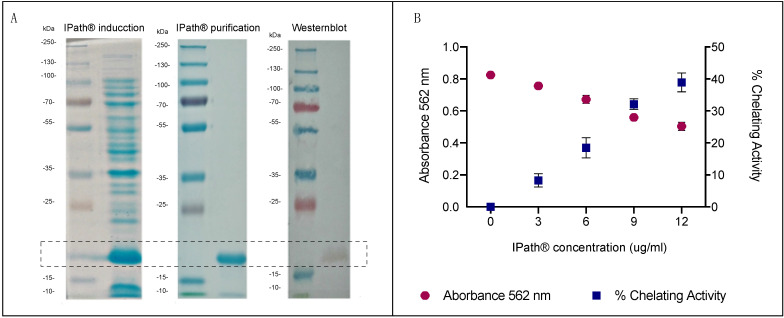
Purification and chelating activity of recombinant IPath^®^ protein. (**A**) SDS-PAGE (12%) analysis of protein extraction products; SDS-PAGE (12%) analysis of purified IPath^®^ by size exclusion chromatography; Western blot analysis of purified IPath^®^ with an anti-His antibody. (**B**) Iron chelating activity of purified recombinant IPath^®^.

IPath^®^ was cloned into a bacterial expression vector to obtain a purified protein of 21 kDa (Figure 1A). The expressed IPath^®^ showed iron-chelating activity based on color reduction of the iron–ferrozine complex (Figure 1B). Average values at 562 nm for negative and positive control were 0.824 and 0.245, respectively. Chelating activity was determinate at different IPath^®^ concentration 1, 3, 6, 9, and 12 μg/mL, obtaining 0.49%, 7.17%, 20.15%, 31.04%, and 40.38% of iron-chelating activity, respectively.

### 3.2. Modulation of IPath^®^ Vaccine in Atlantic Salmon Genes

**Figure 2:**
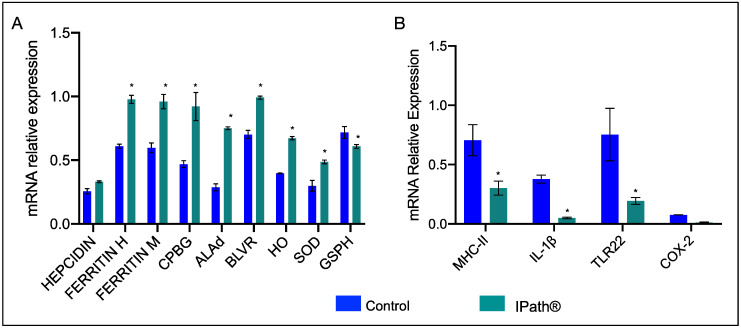
RT-qPCR of (**A**) iron homeostasis and (**B**) immune response-related genes in head kidney of Atlantic salmon injected with IPath^®^ and control group after 400 accumulated thermal units (ATUs). * Indicates significant differences between IPath^®^-vaccinated Atlantic salmon and the control group (*p* < 0.005).

**Figure 3:**
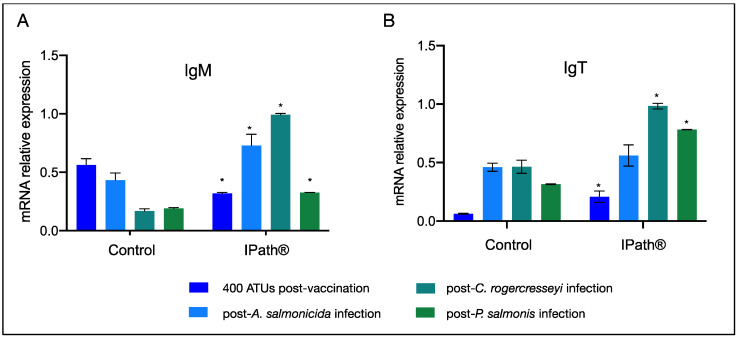
RT-qPCR of (**A**) *IgM* and (**B**) *IgT* of blood cells of Atlantic salmon injected with IPath^®^ and control group. * Indicates significant differences between IPath^®^-vaccinated Atlantic salmon and the control group (*p* < 0.005).

Expression changes of iron-homeostasis-related and immune response genes were evaluated in Atlantic salmon after immunization with IPath^®^ and the control formulation. Overall, Atlantic salmon vaccinated with IPath^®^ showed upregulation of ferritin genes, both chain heavy and medium, and genes associated with heme biosynthesis and degradation compared with the control fish groups (Figure 2A). However, the transcription of immune-related genes showed downregulation in fish immunized with IPath^®^ (Figure 2B). Notably, the evaluation at the transcriptional level of immunoglobulin T (*IgT)* evidenced an increasing activity in immunized Atlantic salmon pre-challenge with pathogens compared to that in the control group (Figure 3). 

### 3.3. IPath^®^ Vaccine Effects in Atlantic Salmon’s Response to Pathogen Infection

*IgM* and *IgT* gene expression profiles were evaluated in the blood cells of immunized Atlantic salmon challenged with different pathogens (Figure 3). The salmon group exposed to *A. salmonicida* infection showed a significant upregulation of *IgM* in IPath^®^-vaccinated Atlantic salmon comparing with the control salmon group (Figure 3A). On the other hand, immunized Atlantic salmon exposed to infection by the ectoparasite *C. rogercresseyi* showed highly expressed *IgM* and *IgT* genes in comparison with the control group (Figure 3A,B). Fish infected with sea lice and subsequently challenged with *P. salmonis* showed upregulation of *IgM* and *IgT* genes in salmons injected with IPath^®^. However, *IgM* and *IgT* expression levels decreased during the *P. salmonis* infection and after the sea lice challenge (Figure 3A,B). 

**Figure 4:**
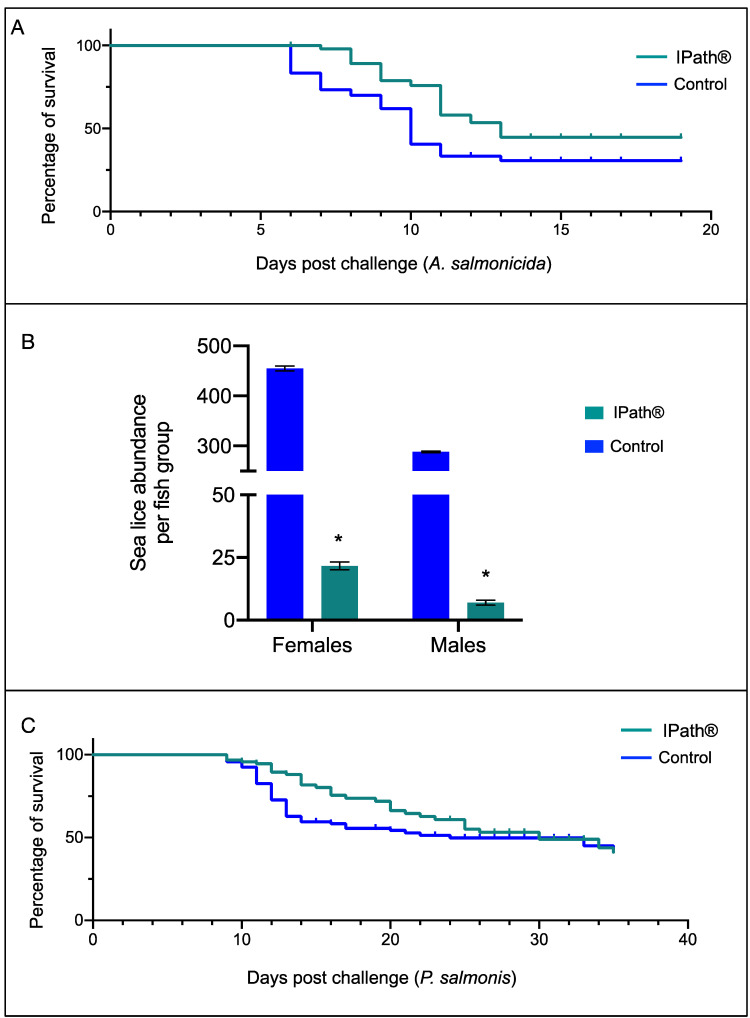
Survival and sea lice burden in Atlantic salmon vaccinated with IPath^®^. (**A**) Probability of Atlantic salmon survival on *Aeromonas salmonicida* infection. (**B**) *Caligus rogercresseyi* abundance per fish group. * Indicates significant differences between IPath^®^-vaccinated fish and the control group (*p* < 0.005). (**C**) Probability of Atlantic salmon survival on *P. salmonis* infection.

Furthermore, vaccinated Atlantic salmon showed a significant reduction of infection risk comparing with the control groups. For instance, during *A. salmonicida* infection, the IPath^®^-vaccinated group showed two days of delay in the mortality peak in relation to the control group (Figure 4A). Furthermore, significant sea lice burden reduction was recorded in vaccinated Atlantic salmon, with an average sea lice burden of 17 in vaccinated fish compared with an average burden of 407 adult sea lice in the control fish group (Figure 4B). Similar results were observed for *A. salmonicida*, where Atlantic salmons vaccinated with IPath^®^ showed a mortality delay during *P. salmonis* infection compared with the control group (Figure 4C).

### 3.4. IPath^®^-Vaccinated Atlantic Salmon Transcription Expression during the Pathogen Infections

**Figure 5:**
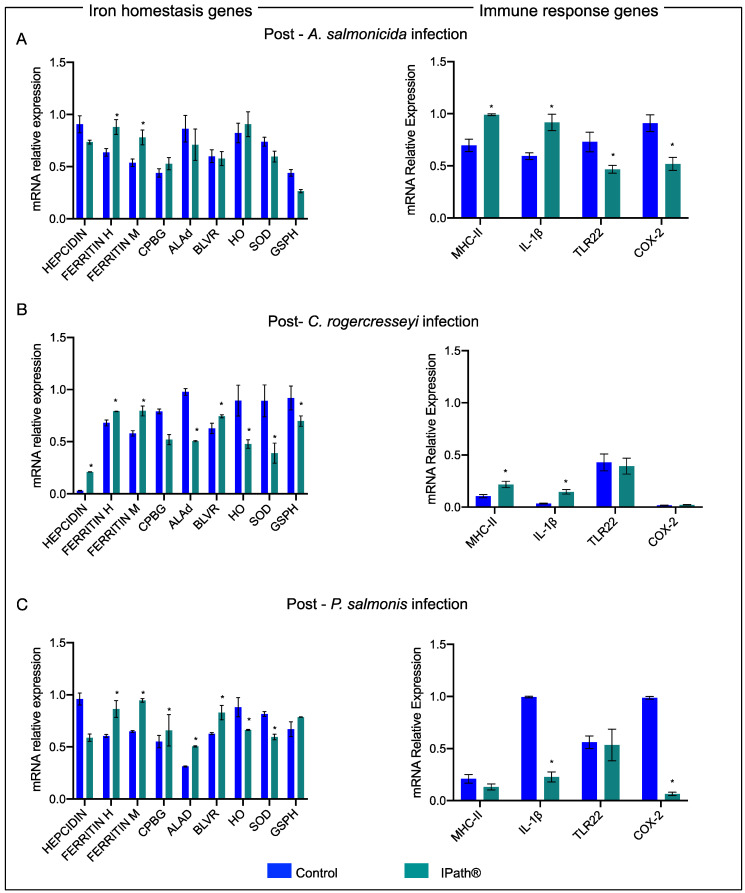
Transcription expression profiles of iron-homeostasis- and immune-related genes in the head kidney of Atlantic salmon immunized with IPath^®^ and control group after pathogen challenges. (**A**) *A. salmonicida* infection response. (**B**) *C. rogercresseyi* infection response. (**C**) *P. salmonis* infection response. * Indicates significant differences between IPath^®^-vaccinated Atlantic salmon and control group (*p* < 0.005).

Transcription expression profiles were analyzed from immunized Atlantic salmons experimentally infected with *A. salmonicida.* Here, iron-homeostasis- and immune-related genes in the head kidney tissue were evaluated. The results evidenced that the *ferritin* gene was upregulated in vaccinated fish groups during the infection with *A. salmonicida* compared with that in the control group. Moreover, genes associated with ROS responses, such as *SOD* and *GSPH*, showed downregulation in immunized fish in response to *A. salmonicida.* The immune response evidenced that *Il-1β* and *MHCII* genes were upregulated in response to the bacterial infection in fish groups immunized with IPath^®^ compared to the control group (Figure 5A). 

Moreover, similar results were observed in the head kidney tissue of Atlantic salmon infected with *C. rogercresseyi*. The ferritin transcripts were significantly upregulated in IPath^®^-vaccinated Atlantic salmon compared with the control group. The *hepcidin* gene and *BLVR* gene, associated with heme biosynthesis, showed high expression levels in immunized salmon during sea lice infection (Figure 5B). The immune response genes *MHCII* and *Il-1β* of vaccinated Atlantic salmon showed increasing transcription levels during sea lice infection. Moreover, the *TLR22* gene, associated with sea lice infection, evidenced similar expression levels in both salmon groups (Figure 5B). The vaccinated Atlantic salmon groups, previously infected with the sea lice, and subsequently infected with *P. salmonis*, showed significant transcription expression changes of iron-homeostasis-related genes such as ferritin and heme biosynthesis genes (*CPBG*, *ALAd*, *BLVR*) (Figure 5C). However, the immune response genes were observed to be downregulated compared with the control group.

**Figure 6:**
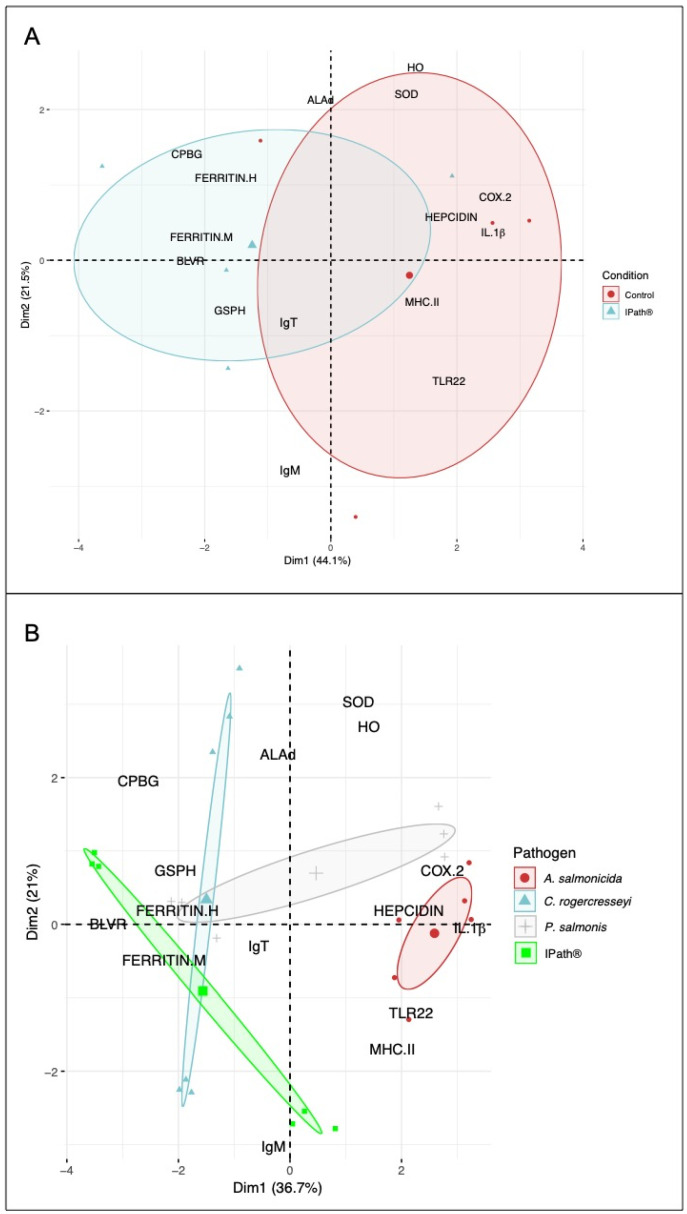
Principal component analysis for transcription expression of iron-homeostasis- and immune-related genes in Atlantic salmon. (**A**) Biplot based on experimental groups (e.g., IPath^®^ vaccine vs. control group) as a factor for gene expression differences. (**B**) Biplot based on pathogen challenge (e.g., *A. salmonis* infection, *C. rogercresseyi* infection, *P. salmonis* infection) as a factor for gene expression differences. Both biplots were constructed based on gene expression analyses obtained by qPCR reactions.

Finally, PCA analysis was conducted using the expression levels of iron-homeostasis- and immune-related genes. Two statistical comparisons were performed. The first one evaluated the effect of IPath^®^ vaccination in the transcription activity of Atlantic salmon. The analysis showed a high tendency of iron-homeostasis-related gene modulation after 400 ATUs in fish groups immunized with IPath^®^ compared with that in the control group (Figure 6A). Furthermore, *IgT* transcription expression showed association with vaccinated Atlantic salmon (Figure 6B). The second analysis was conducted to explore how pathogens can modulate gene expression during the infection process. The PCA showed a relationship with iron homeostasis genes in *C. rogercresseyi* and *P. salmonis* infection (Figure 6B). In contrast, the immune-related genes were associated with *A. salmonicida* infection. 

## 4. Discussion

Due to the importance of iron homeostasis, organisms have evolved to develop different mechanisms to cope with iron fluctuations. In vertebrates, the iron excess is controlled by proteins with the capacity to couple to iron. Some of them include haptoglobin, ferritin, transferrin, and hepcidin [41,42,43]. Among them, hepcidin plays a pivotal role in the regulation of iron homeostasis, fulfilling roles in the regulation of iron concentrations in plasma, tissue distribution of iron in the intestine, iron storage in the liver, and iron recycling by macrophages [44,45]. Hepcidin acts as a signaling molecule for iron storage in macrophages in mammals [45,46]. Hepcidin is also produced in response to inflammatory stimuli. Furthermore, in humans, it is possible to associate high levels of hepcidin to iron overload and inflammatory stages [47]. Fish are also affected by abnormal iron concentrations at a systemic level. For instance, Atlantic salmon and rainbow trout exposed to acute iron doses showed a reduction of hemoglobin levels, suggesting severe erythrocyte damage [48]. Moreover, hematological changes were observed in *Labeo rohita* after exposure to high levels of iron, having repercussions in the respiratory function, damaging tissues, and increasing leukocytes and hemoglobin levels [49]. Furthermore, the authors suggest a pathological change in liver tissue as a consequence of iron overload [49]. Due to the importance of iron in fish health, iron has been supplemented in commercial diets to promote the productive performance of fish. However, a study performed in Atlantic salmon demonstrates that high supplementation of iron in fish’s diets can increase their susceptibility to bacterial disease [50].

Iron is an essential element for pathogen proliferation during an infective process. Expression levels changes of iron-homeostasis-related genes have been reported in Atlantic salmon during bacterial and ectoparasite infections [15,17,35,36]. Moreover, different molecular mechanisms of the intracellular bacterium *P. salmonis* for host iron acquisition have been reported [36,51]. Furthermore, upregulation of the ferritin gene during Atlantic salmon infection with the ectoparasite *C. rogercresseyi* has been observed [52]. In this way, the mechanism for iron regulation and homeostasis is a vital strategy for fish in response to pathogens. 

The engineered recombinant chimeric protein (IPath^®^) exhibits a chelator activity, consistent with the iron-binding quality of proteins related to iron metabolism. This fact evidences a putative biological functional activity related to iron regulation. Furthermore, iron-homeostasis-related genes in Atlantic salmon vaccinated with IPath^®^ after 400 ATUs showed an increased expression level. Therein is suggested an iron concentration variation in Atlantic salmon due to injection with IPath^®^ that triggers an adverse environment for pathogen infection. The impact on pathogen infection of the chelating activity of recombinant iron transport proteins has been reported in fish. For instance, *L. anguillarum* growth inhibition has been reported during exposure to recombinant ferritin characterized from turbot (*Scophthalmus maximus*) [20]. The authors suggest that the recombinant’s chelating function reduces iron molecules available for the bacterial [20]. Antibacterial and antiviral resistance has also been reported in turbot exposed to the administration of two isoforms of hepcidin [21]. In this sense, our results suggested a protective effect of IPath^®^ as a candidate vaccine in Atlantic salmon exposed to *A. salmonicida* and *P. salmonis* infection. Furthermore, a sea lice burden reduction also was observed. *C. rogercresseyi* females obtained from IPath^®^-vaccinated Atlantic salmon also exhibited egg strain damage and reduced eggs number, suggesting an adverse effect of the IPath^®^ vaccine in sea louse reproductive output. Similarly, an observation was reported in rabbits injected with recombinant ferritin, where tick burden and egg viability reduction were observed [23]. These findings suggest that the iron-chelating proteins impact the iron homeostasis in parasites with blood-feeding behavior. Unlike most eukaryotes, hematophagous organisms possess an incomplete heme biosynthetic pathway [53]. Here, dietary hemoglobin has been reported as an exogenous source of heme distributed by hemolymph carrier proteins and sequestered by vitellins in the development of oocytes and further embryogenesis [54].

It is known that iron concentration has an influence on the immune process, such as the inflammatory response, nitric oxide formation, immune cell differentiation, and stress response, increasing ROS production [55,56,57,58,59]. For instance, in mammals, an increase of *TNF-alpha* gene expression has been observed to response to a reduction in iron levels [60]. The inhibition of *IFN-gamma* expression has also been reported in the presence of high concentrations of iron [61]. In this study, the protective effect of IPath^®^ was evaluated in two different infection trials. In the first one, after 400 ATUs, Atlantic salmon were vaccinated with IPath^®^ against the bacterium *A. salmonicida*. Unlike reported in sea bass larvae exposed to recombinant ferritin and subsequently infected with *Vibrio anguillarum* [22], IPath^®^-vaccinated fish groups showed high activity of *MCHII* and *IL-1β* genes in response to *A. salmonicida*. Among the immune genes associated with fish response to *C. rogercresseyi*, *MHCII* and *TLR22* genes have been associated with inflammatory responses [62]. Here, immunized Atlantic salmon showed upregulation of *MCHII* and *IL-1β* genes compared with the control group, suggesting an immune-modulation response to IPath^®^ vaccination. This study also showed upregulation of *IgM* and *IgT* genes in IPath^®^-vaccinated Atlantic salmon after *A. salmonicida*, *C. rogercresseyi*, and *P. salmonis* infections. The analysis suggests an Ig positive regulatory effect of recombinant iron-related proteins. Unlike in mammals, suppression of Ig production in peripheral blood mononuclear cells associated with iron regulation in monocytes has been reported [63]. 

Related to ferritin expression, the induction of this gene in response to bacterial infection has been reported in different fish species, for example, in turbot [20], sea bass [5], and Atlantic cod [64]. Furthermore, ferritin and hepcidin upregulation has been reported in Atlantic salmon vaccinated with *A. salmonicida* genetically attenuated strain [65]. Upregulation of transferrin transcripts has also been reported in Atlantic salmon during *A. salmonicida* infection [66]. Here, an upregulation of ferritin transcripts in IPath^®^-immunized Atlantic salmon infected with *A. salmonicida* compared with unvaccinated fish was observed. *A. salmonicida* has an efficient host-iron acquisition mechanism for a successful infection [67]. It is possible to suggest that an increase in iron store activity triggered by the IPath^®^ vaccine reduces the iron available to *A. salmonis*, reflecting in a reduction of Atlantic salmon’s susceptibility to the pathogen.

From the second trial, iron homeostasis gene regulation was evaluated in vaccinated Atlantic salmon infected with *C. rogercresseyi*. The transcription analysis showed an increased expression level of iron homeostasis genes in immunized fish after sea lice infection compared with the control. The upmodulation of iron homeostasis genes favors a nutritional immunity strategy. This response has been reported in the species Coho salmon, which is naturally resistant to *C. rogercresseyi* infections [17]. Furthermore, Atlantic salmon infected with the sea lice species *L. salmonis* and *C. rogercresseyi* reveal that the nutritional immunity response seems highly relevant to face ectoparasites [16]. The protective effects of IPath^®^ in Atlantic salmon during a secondary infection with *P. salmonis* were evaluated. Although the final survival fraction was similar between both groups, a significant mortality delay was recorded in vaccinated groups compared with control. The reduced sea lice burden could explain this dynamic in IPath^®^-immunized fish, and the up-modulation of iron homeostasis genes observed in the vaccinated Atlantic salmon. Previous research has highlighted the importance of the iron regulatory mechanism in Atlantic salmon’s response to *P. salmonis* infection [36,67,68]. These findings suggest that susceptible individuals trigger intracellular iron storage and putatively regulated it through the heme biosynthesis/degradation pathways [36]. In general, high transcription expression of iron-related genes in vaccinated fish suggests a protective effect of chimeric iron-related proteins. Finally, the PCA analysis suggests a positive modulation of iron-homeostasis-related genes, where ferritin is highly activated in Atlantic salmon immunized with IPath^®^.

## 5. Conclusions

This study showed the effects of an engineered recombinant protein related to iron metabolism in Atlantic salmon. IPath^®^ evidences the capacity to modulate the iron homeostasis and immune response in Atlantic salmon during the bacterial and ectoparasite infection processes. From the PCA analysis, it is possible to determine that IPath^®^ influences Atlantic salmon iron-homeostasis genes. Based on this, we hypothesize that IPath^®^ induces changes in host iron homeostasis mechanisms that indirectly generate a protective effect in the host against infections. Notably, mortality reduction in response to *A. salmonicida* and *P. salmonis* and in the sea lice burden was observed in IPath^®^-vaccinated groups. Cumulatively, the novel chimeric iron-related protein named IPath^®^ is a candidate vaccine for commercial testing in the salmon farming aquaculture. 

## Figures and Tables

**Table 1:** Primer list used for RT-qPCR analysis.

Primer Name	Gene	Sequence 5′-3′	Tm	Efficiency (%)
EF1_F2	*Elongation factor* (reference gene)	TGCTGGTGGTGTGGGTGAGT	60	95.18
EF1_R2		CCTCAAACCGCTTCTGGCTGT		
IgM_F	*Immunoglobulin M*	TGAGGAGAACTGTGGGCTACACT	60	99.98
IgM_R		TGTTAATGACCACTGAATGTGCAT		
IgM_Probe		CATCAGATGCAGGTCC		
IgT_F	*Immunoglobulin T*	CAACACTGACTGGAACAACAAGGT	60	107.77
IgT_R		CGTCAGCGGTTCTGTTTTGGA		
IgT_Probe		AGTACAGCTGTGTGGTGCA		
IL1b_F6	*Interleukin-1b*	GATCTGGAGGTATCCCATCA	60	122.36
IL1b _R6		CACAGCACTCTCCAGCAAGA		
COX-2_F	*Cyclooxygenase 2*	CAGTGCTCCCAGATGCCAAG	60	102.34
COX-2_R		GCGAAGAAGGCGAACATGAG		
TLR22_3_F	*Toll-like receptor 22*	TGCCTTCTAACCCTCTCCCT	61	91.36
TLR22_3_R		CGCTGCTCTCAGACAGGAAG		
MHCII_F	*Major histocompatibility complex II*	CTCCTCAAAGGACCTGCAGG	60	104.72
MHCII_R		TCAGGACCTTTGTTCCAGGC		
GSHPx_F1	*Glutathione peroxidase*	TAAAGTGGTGCTGATCGAGA	54	100.23
GSHPx_R1		GTTCTCCTGATGTCCGAACT		
SOD_F1	*Superoxide dismutase*	CCGTATTCTTTGAGCAGGAG	54	104.98
SOD_R1		AGCCGTTGGTGTTGTCTC		
ALA d_F2	*Aminolevulinate dehydratase*	CCACTCGCCCATCCATCATA	59	126.72
ALA d_R2		ACACCTCACATGGACACTGT		
ALA s_F1	*Aminolevulinate synthase*	GGTAGGATGCCTGCTGACTG	63	105.52
ALA s_R1		CCCCAAGCCTGTTTTGCTGA		
HO_F2	*Heme oxygenase*	GTCCTCTCGAGTGGTGAAGC	61	94.21
HO_R2		ATCTCTGAGTCCCTGGCCAA		
Blv r_F1	*Biliverdin reductase*	AAACAGATCCACCAGCCAGG	59	106.7
Blv r_R1		ACAGCCGACTTTAAGCAGCT		
Hep_F1	*Hepcidin*	GCTGTTCCTTTCTCCGAGGTGC	59	111.07
Hep_R1		GTGACAGCAGTTGCAGCACCA		
FerritinM_F1	*Ferritin M*	TATCACCACGATTGCGAAGC	60	102.66
FerritinM_R1		CTCGTCGCTGTTCTCCTTGA		

## Data Availability

This study did not report any data.
